# Supplementation of enteral nutritional powder decreases surgical site infection, prosthetic joint infection, and readmission after hip arthroplasty in geriatric femoral neck fracture with hypoalbuminemia

**DOI:** 10.1186/s13018-019-1343-2

**Published:** 2019-09-03

**Authors:** Yaoquan He, Jun Xiao, Zhanjun Shi, Jinwen He, Tao Li

**Affiliations:** 1 Department of Orthopedics, Nanfang Hospital, Southern Medical University, Guangzhou, Guangdong China; 2 Department of Rehabilitation, Nanfang Hospital, Southern Medical University, Guangzhou, Guangdong China

**Keywords:** Hip arthroplasty, Hypoalbuminemia, Nutritional supplementation, Surgical site infection, Periprosthetic joint infection

## Abstract

**Background:**

Nearly half of elderly patients with hip fracture were malnourished, indicated with a serum marker of hypoalbuminemia. Malnutrition was a risk factor for poor outcomes in geriatrics after hip replacement. The purpose of this study was to investigate if oral nutritional supplementation after the procedure in geriatrics with hypoalbuminemia was beneficial for outcomes.

**Methods:**

A retrospective cohort study of older (≥ 65 years old) patients suffering femoral neck fracture and undergoing hip replacement with hypoalbuminemia was conducted. Outcomes were compared between patients with and without postoperative nutritional supplementation.

**Results:**

There were 306 geriatric patients met the criteria. Following adjustment for baseline characteristics, patients with nutritional supplementation showed a lower grade of wound effusion with adjusted OR 0.57 (95% confidence interval (CI), 0.36 to 0.91, *P* < 0.05). And also a lower rate of surgical site infection (5.5% compared with 13.0% [adjusted OR 0.40, 95% CI, 0.17 to 0.91, *P* < 0.05]), periprosthetic joint infection (2.8% compared with 9.9% [adjusted OR 0.26, 95% CI, 0.08 to 0.79, *P* < 0.05]), and 30 days readmission (2.1% compared with 8.7% [adjusted OR 0.22, 95% CI, 0.06 to 0.79, *P* < 0.05]). The average total hospital stay was longer in patients without nutritional supplementation (10.7 ± 2.0 compared with 9.2 ± 1.8 days, *P* < 0.05).

**Conclusions:**

The data suggest that postoperative nutritional supplementation is a protective factor for surgical site infection, periprosthetic joint infection, and 30-days readmission in geriatric with hypoalbuminemia undergoing a hip replacement. Postoperative nutritional supplementation for these patients should be recommended.

**Electronic supplementary material:**

The online version of this article (10.1186/s13018-019-1343-2) contains supplementary material, which is available to authorized users.

## Background

Hip arthroplasty (HA), including total hip arthroplasty and semi-hip arthroplasty, is one of the most common surgical procedures performed in geriatric femoral neck fracture. Although HA is a safe, effective procedure, a small percentage of HA procedures do result in major complications, including pneumonia, surgical site infection (SSI), wound dehiscence, prosthetic joint infection (PJI), heart failure, and death [1–4]. Prior studies have identified respiratory disease, older age, diabetes mellitus, and prior infection as risk factors for postoperative complications [1, 5–8].

Malnutrition has been identified as another potential risk factor for poor outcomes. It has been shown to increase the risk of SSI, wound dehiscence, and PJI [4, 9, 10]. Unfortunately, it has been shown that more than half of in-hospital geriatric patients present with malnutrition [11]. Hypoalbuminemia (serum albumin concentration < 3.5 g/dL) is most commonly used serum marker of malnutrition [12]. It has shown that the percentage of hypoalbuminemia in elderly patients with hip fracture is nearly 50% [13, 14].

Although hypoalbuminemia is potentially modifiable before the operation, it is difficult to achieve for a limited time for the purpose of reducing complications (such as pneumonia, deep vine thrombosis) due to prolonged bed rest in geriatric femoral neck fracture patients. Current nutrition guidelines state that oral nutritional supplements are recommended in geriatric patients after hip fracture and orthopedic surgery to reduce complications [14–16]. However, contradictory results have found. Several studies, including RCT (randomized placebo-controlled test) [17], indicated beneficial effects of nutritional status, hospital stay, clinical outcome, and mortality [17–20]. Other studies failed to demonstrate these [19, 21]. A recent meta-analysis included 41 trials and 3881 participants, the results showed low-quality evidence of nutritional supplementation on complications and mortality [22].

The heterogeneity of included patients and surgical procedures may be a significant limitation to delineate accurate conclusion [22]. The purpose of this study was to retrospectively investigate the effect of oral supplementation of enteral nutritional powder on the complications of geriatric femoral neck fracture with hypoalbuminemia. We analyzed the effect of nutritional supplementation on a particular cohort to decrease heterogeneity.

## Methods

### Inclusion and exclusion

We conducted a retrospective cohort of older patients (≥ 65 years of age) with hypoalbuminemia (serum albumin concentration < 3.5 g/dL) who were admitted to our hospital between February 2007 and February 2017 because of femoral neck fracture and HA was the treatment of choice. Patients with moderate to severe malnutrition (weight loss of > 5% in the previous month or > 10% in the previous 6 months and/or serum albumin concentration < 2.7 g/dL) were excluded. Other exclusion criteria were severe heart failure (New York Heart Association class III or IV), respiratory failure, acute and/or chronic renal failure, hepatic insufficiency or cirrhosis (Child B or C), and any of gastrointestinal or cognitive condition that may preclude the patient from adequate oral nutrition intake. This study was approved by the Regional Ethics Committee of our hospital.

The cohort was divided into two groups by the treatment of oral nutrition supplementation, Enteral Nutritional Powder (TP, ENSURE®), which started around February 2014.

### Data elements

#### Interventions

The cohort received a surgical procedure of HA at days 1–3 after admission and a 12-h overnight fast. The surgeries were conducted by four different surgeons in the same procedure, which was under spinal anesthesia with supine position and lateral approach. Anticoagulation, analgesic and rehydration therapy was routinely conducted postoperatively. For hypoalbuminemia (serum albumin concentration < 3.5 g/dL), all the patients were treated with one of the solutions after surgery. The control group was treated with intravenous infusion of human albumin only. And the nutritional supplementation group was treated with Enteral Nutritional Powder (TP, ENSURE®). At POD3 (post operation day 3), blood test was carried out to check whether hypoalbuminemia was corrected (serum albumin ≥ 3.5 g/dl). If yes, the treatments stopped. If no, Enteral Nutritional Powder (TP, ENSURE®) plus intravenous infusion of human albumin was the new solution for the nutritional supplementation group. For both groups, treatment stopped only when hypoalbuminemia was corrected (serum albumin ≥ 3.5 g/dl) or wound effusion disappeared. Enteral Nutritional Powder (TP, ENSURE®) was given at a dose of 250 ml (200 ml cold water added with 55.8 g ENSURE® powder, providing 251.1 kcal and 8.87 g protein. The energy distribution was 14.2% protein, 54% carbohydrate and 31.8% fat) by 3 times a day. A detailed formula of Enteral Nutritional Powder (TP, ENSURE®) was shown in Additional file 1: Table S1. The cohort received the same rehabilitations postoperative.

#### Data collection

Patient variables included demographics (age and gender), body mass index (BMI), and preoperative comorbidities (hypertension requiring medication, diabetes mellitus, congestive heart failure, chronic obstructive pulmonary disease, chronic renal failure, hepatitis). Laboratory indicators included serum albumin, total lymphocyte count (TLC), hemoglobin, and blood urea nitrogen (BUN) preoperative, and postoperative. We also documented the combined medication such as glucose, amino acids, fat emulsion, human serum albumin, and blood transfusion.

#### Outcomes

The primary outcomes included rates of major and minor postoperative complications, such as SSI, wound effusion, wound complications (including wound dehiscence, delayed healing, edema, and hematoma), and PJI. Secondary outcomes included 30-day readmission and reoperation rates, total hospital length of stay, and total amount of human albumin intravenously infused.

When considering wound effusion, we counted the number of infiltrated sterile gauze, which was routinely covering the wound after operation. We usually use four pieces of sterile gauze to cover the wound. Any infiltration over four pieces was documented as “large amount of wound effusion” and were defined as “5” in the analysis. For convenience for analyses, we defined 0 and 1 as “little” effusion, 2 and 3 as “moderate” effusion, and 4 and 5 as “large” effusion. We also evaluated functional outcome using the Harris hip scores at 1 month follow-up.

### Statistical analyses

Statistical analyses were conducted in SPSS Statistics 22. The level of significance was set at α = 0.05 (*P* < 0.05) and all the tests were two-tailed. Results of continuous variables were expressed as means±SD, and frequency and ratio were used for categorical or ordinal variables. Shapiro-Wilk tests were used to investigate a normal distribution. Comparisons between the two groups at baseline and outcome were performed using one-way ANOVA and independent *t* test for continuous variables with normal distribution, and chi-square test or Fisher exact test for discontinuous variables. Mann-Whitney *U* tests were used for non-normal distributed variables. In each of these analyses, missing values were excluded if they were less than 5%.

Finally, logistic regression analysis was performed to calculate adjusted odds ratios (ORs) with 95% confidence intervals (CIs) for the association between oral nutrition supplementation and outcomes. Only preoperative variables found to be statistically associated with postoperative outcomes were included in the logistic regression analysis.

## Results

From February 2007 to February 2017, there were 782 patients admitted in our hospital due to femoral neck fracture and HA was the treatment. Three hundred fifty-six were excluded due to age (younger than 65 years old). And then 114 were excluded due to normal serum albumin (≥ 3.5 g/dL). Six were excluded due to severe hypoalbuminemia (4 for serum albumin < 2.8 g/dL) or comorbidities (1 for end stage of chronic renal failure, 1 for cognitive dysfunction, no others were found). Finally, a total of 306 patients were conducted into our cohort, 145 in the nutritional supplementation group, and 161 in the control group. Almost all of these cases were caused by falls (low-energy damage). Only three of them were caused by traffic accident (high-energy damage), 1 in the nutritional supplementation group and 2 in the control. Basal characteristics of the patients were shown in Table 1. No statistically significant difference was found between these two groups. All the primary and secondary outcomes were shown in Table 2.

**Table 1:** Clinical and biochemical characteristics of included patients at baseline

	Control	Nutr. supple.	Statistics
Gender, male/%	71/44.1	68/46.9	*χ*^*2*^ = 0.24, *P* = 0.62
Age, years	78.2 ± 6.7	77.6 ± 6.0	*t* = − 1.80, *P* = 0.07
Weight, kg	56.50 ± 8.70	58.25 ± 8.30	*t* = − 1.57, *P* = 0.12
Body mass index, kg/m^2^	21.3 ± 2.0	21.6 ± 2.1	*t* = 0.85, *P* = 0.39
Serum albumin concentration, g/dL	3.24 ± 0.12	3.23 ± 0.12	*t* = 0.67, *P* = 0.51
Total lymphocyte count (TLC), /dL	1463 ± 171	1481 ± 162	*t* = − 0.93, *P* = 0.35
Hemoglobin, g/L	118.3 ± 11.3	118.9 ± 11.1	*t* = − 0.46, *P* = 0.65
Blood urea nitrogen, mmol/L	5.3 ± 1.56	5.4 ± 1.4	*t* = − 0.52, *P* = 0.60
Comorbidities			
Hypertension, *n*/%	19/11.8	20/13.8	*χ*^*2*^ = 0.27, *P* = 0.60
Diabetes mellitus, *n*/%	10/6.2	16/11	*χ*^*2*^ = 2.28, *P* = 0.13
Congestive heart failure, *n*/%	4/2.5	4/2.8	*χ*^*2*^ = 0^a^, *P* = 1
COPD, *n*/%	4/2.5	2/1.4	*χ*^*2*^ = 0.08^a^, *P* = 0.78
Chronic renal failure, *n*/%	2/1.2	2/1.4	*χ*^*2*^ = 0 ^a^, *P* = 1
Hepatitis, *n*/%	1/0.6	0	*P* = 1^b^
Others, *n*/%	0	0	–

*Nutr. supple.* nutritional supplementation; *COPD* chronic obstructive pulmonary disease

^a^Continuity correction

^b^Fisher’s exact test

**Table 2:** Postoperative characteristics

	Control	Nutri. supple.	Statistics
Maximum Hb drop, g/L	31.4 ± 4.1	30.3 ± 4.1	*t* = 2.44, *P* = 0.01
Amount of ALB infusion, g	61 ± 22	44 ± 18	M–W *U* = 6384.5, *P*< 0.001
Amount of wound effusion			
Little (0,1), *n*/%	51/32	65/45	M–W *U* = 9627.5, *P* = 0.005
Moderate (2,3), *n*/%	45/28	42/29	
Large (4,5), *n*/%	65/40	38/26	
Complications			
wound complication, *n*/%	27/16.8	11/7.6	*χ*^*2*^ = 5.92, *P* = 0.02
SSI, *n*/%	21/13.0	8/5.5	*χ*^*2*^ = 5.04, *P* = 0.03
PJI, *n*/%	16/9.9	4/2.8	*χ*^*2*^ = 6.44, *P* = 0.01
Nausea, *n*/%	29/18.0	23/15.9	*χ*^*2*^ = 0.25, *P* = 0.62
Vomiting, *n*/%	28/17.4	18/12.4	*χ*^*2*^ = 1.48, *P* = 0.22
Diarrhea, *n*/%	5/3.1	6/4.1	*χ*^*2*^ = 0.24, *P* = 0.63
Others, *n*/%	0	0	–
30-day readmission, *n*/%	14/8.7	3/2.1	*χ*^*2*^ = 6.39, *P* = 0.01
Reasons of readmission			
Wound complications, *n*/%	2/1.2	2/1.4	*χ*^*2*^ = 0.15^a^, *P* = 0.69
Dislocation, *n*/%	1/0.6	1/0.7	*χ*^*2*^ = 0.40^a^, *P* = 0.52
Gastrointestinal diseases, *n*/%	8/5.0	0	–
MI, *n*/%	1/0.6	0	–
DVT, *n*/%	1/0.6	0	–
Pneumonia, *n*/%	1/0.6	0	–
30-day reoperation, *n*/%	4/2.5	3/2.1	*P* = 1^b^
Total hospital length of stay, days	10.7 ± 2.0	9.2 ± 1.8	M–W *U* = 6520.0, *P* < 0.001

*Nutr. supple.* nutritional supplementation; *SSI* surgical site infection; *PJI* periprosthetic joint infection; *MI* myocardial infarction; *DVT* deep vein thrombosis

^a^Continuity correction

^b^Fisher’s exact test

### Primary outcomes

Patients with nutritional supplementation showed a lower grade of wound effusion when considering “little” effusion, results indicated with adjusted OR 0.57 (95% CI, 0.36 to 0.91; *χ*^*2*^ = 5.61, *P* = 0.02). Also, patients with nutritional supplementation indicated a lower rate (7.6%) of total wound complications than that in the control (16.8%) with adjusted OR 0.41 (95% CI, 0.19 to 0.86; *χ*^*2*^ = 5.92, *P* = 0.02). The rates of SSI and PJI in the nutritional supplementation group were 5.5% vs. 13.0% and 2.8% vs. 9.9% respectively, indicating a beneficial effect of nutritional supplementation with adjusted OR 0.40 (95% CI, 0.17 to 0.91; *χ*^*2*^ = 5.04, *P* = 0.03) and 0.26 (95% CI, 0.08 to 0.79; *χ*^*2*^ = 6.44, *P* = 0.01) respectively.

### Secondary outcomes

Patients with nutritional supplementation (2.1%) had lower rate of 30-day readmission than patients without (8.7%), with adjusted OR 0.22 (95% CI, 0.06 to 0.79, *χ*^*2*^ = 6.39, *P* = 0.01). The main reason for 30-day readmission was gastrointestinal diseases in the control group (*n* = 8, 5.0%; none was observed in nutritional supplementation group). No statistical difference was observed in concern of wound complications (such as wound dehiscence, delayed healing, edema, or hematoma) and dislocation between these two groups. The rest reasons were myocardial infarction (MI), deep vein thrombosis (DVT), and pneumonia shown in Table 2. The rates of 30-day reoperation were similar between these two groups (2.5% vs. 2.1%, Fisher’s exact test, *P* = 1).

The average total hospital length of stay was lower in patients with nutritional supplementation (9.2 ± 1.8 days) than patients without (10.7 ± 2.0 days, Mann-Whitney *U* test 6520.0, *P* < 0.001). For further investigating the reasons, we used a hospital stay time for longer than 11 days as the cutoff for longer hospital stay time. After investigation of the reasons, the results were shown in Table 3. It showed a statistically significant difference in wound complications (wound dehiscence, delayed healing, edema, and hematoma). No statistical difference was observed in other reasons such as pain, poor rehabilitation, pneumonia, urine retention, dizzy, and unknown.

**Table 3:** Reasons of longer hospital stay length

	Control	Nutri. supple.	Statistics*
Total, *n*	52	17	*χ*^*2*^ = 17.33, *P* < 0.01
Reasons			
Wound complication, *n*/%	27/51.9	2/11.8	*χ*^*2*^ = 6.91, *P* = 0.008
Pain, *n*/%	7/13.5	3/17.6	*χ*^*2*^ < 0.01, *P* = 0.98
Poor rehabilitation, *n*/%	5/9.6	4/23.5	*χ*^*2*^ = 1.13, *P* = 0.29
Pneumonia, *n*/%	2/3.8	1/5.9	*χ*^*2*^ = 0.11, *P* = 0.74
Urine retention, *n*/%	1/1.9	2/11.8	*χ*^*2*^ = 1.09, *P* = 0.30
Dizzy, *n*/%	4/7.7	3/17.6	*χ*^*2*^ = 0.51, *P* = 0.47
Unknown, *n*/%	6/11.5	2/11.8	*χ*^*2*^ = 0.17, *P* = 0.68

*All these statistics were with continuity correction except for the row of total

**Fig. 1:**
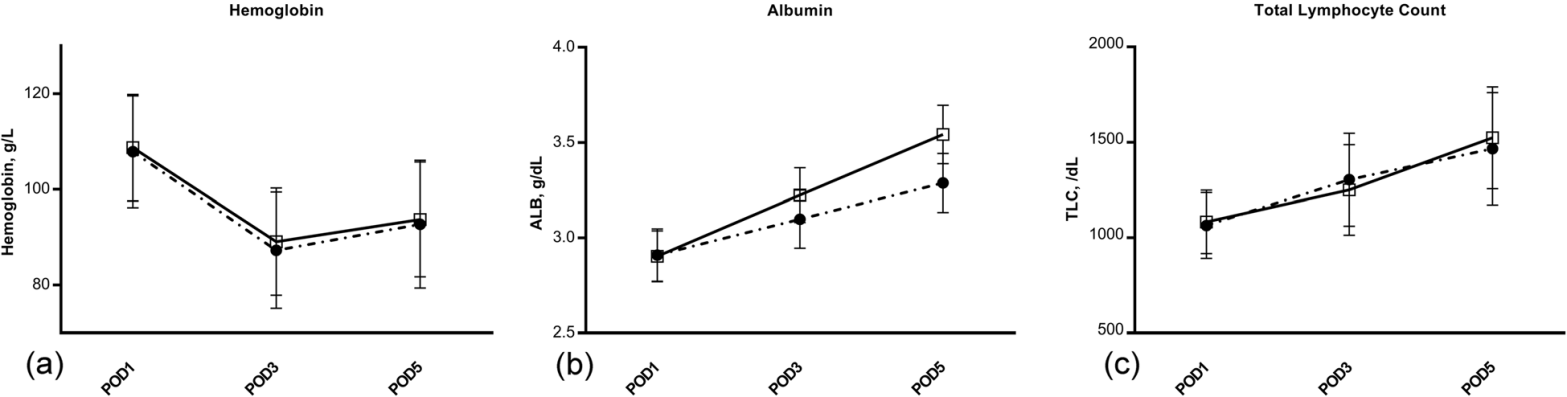
Postoperative hemoglobin, serum albumin concentration, and total lymphocyte count. The symbols represent the means, and error bars show the standard deviation. Filled circles represent controls, whereas open squares represent the nutritional supplementation (TP, ENSURE®, each providing 251.1 kcal and 8.87 g protein, 3 times a day) intervention group. Follow-up data were documented at postoperative day 1 (POD1), day 3 (POD3), and day 5 (POD5). There was a significant difference for serum albumin concentration at POD3 (*t* = − 7.28, *P* < 0.001) and POD5 (*t* = − 14.23, *P* < 0.001). There was a similar change in hemoglobin (*P* < 0.05 at each follow-up) for the two groups at each follow-up. The same was observed for serum total lymphocyte count (*P* < 0.05 at each follow-up)

The amount of total albumin infusion was much lower in patients with nutritional supplementation (44 ± 18g) than that without (61 ± 22 g; Mann-Whitney *U* test 6384.5, *P* < 0.001). When considering serum albumin, there was a similar drop after operation (POD 1) between the 2 groups, but a significant difference was seen at POD 3 (3.22 ± 0.14 g/dL vs. 3.10 ± 0.15 g/dL; *t* = − 7.28, *P* < 0.001) and POD 5 (3.54 ± 0.15 g/dL vs. 3.29 ± 0.16 g/dL; *t* = − 14.23, *P* < 0.001, Fig. 1b). However, the hemoglobin and TLC seemed to be similar in the two groups after operation, independent of nutritional supplementation (Fig. 1a, c).

In terms of the functional outcomes, the average Harris scale at the 1-month follow-up was 83.12 ± 8.75 ranging from 77 to 86 in the nutritional supplementation group, and 82.42 ± 9.15 ranging from 74 to 87 in the control group. No statistically significant difference was observed.

## Discussion

The overall results of the current investigation suggested that nutritional powder supplementation after HA in geriatrics with hypoalbuminemia decreased SSI, PJI, and 30-day readmission. It also seemed to show positive effects on shortening hospitalization and reducing human serum albumin infusion.

Several lines of evidence suggested the relationship between nutritional supplementation and orthopedic surgical outcomes. The results of these studies seemed to be contradictive. Some studies found a beneficial effect on hospital stay [23, 24], and postoperative complications [23–26], other studies failed to demonstrate so [27–29]. One explanation of the discrepancy might be the heterogeneity of included patients, baseline nutritional status, surgical procedures, and nutritional formula [22]. Our study retrospectively reviewed a special HA cohort with similar baseline nutritional status and conducted with the same surgical procedures and nutritional formula except for nutritional supplementation. It is hard to handle a similar study in a conventional HA cohort. Because HA was usually an elective surgery for a conventional cohort, there existed the opportunity to delay the surgery to modify nutritional status. In contrast, the surgery could not be delayed due to nutritional optimization for femoral neck fracture in older patients [18, 20, 30]. Thus, for the special cohort we were reviewed, hypoalbuminemia seemed to be an unmodifiable risk factor and persisted throughout the perioperative period.

As shown in our study, there was a statistically significant improvement in the serum albumin level in the nutritional supplementation group at day 3 postoperation. Serum albumin is a nutritional marker helping patient recovery and benefits the elderly to maintain the immunological functions [31]. Previous studies showed that patients with hypoalbuminemia were likely to suffer from global malnutrition and lack of important proteins for proper immune function [26]. Evidence indicated that nutritional supplementation seemed to improve nutritional status after joint arthroplasty [20, 32]. However, the actual effect of oral nutritional supplementation on the serum albumin level was still unknown. While the earlier study had found a beneficial effect on nutritional status [20], a recent study (Botella-Carretero JI et al.) found no effect during in-hospital follow-up [28]. Adherence to oral nutritional supplementation might be one of the explanations. In the study of Botella-Carretero JI [28], only about half of the supplementation amount was taken. In fact, surgical procedure, anesthesia, nutritional formula, comorbidities, and combination therapy could also be the reason for poor adherence. Given the fact of small size of these studies, the actual effect of nutritional supplementation on serum albumin was not yet certain. In our study, all the patients took the prescribed amount of supplementation. Given the specialty of this cohort, the results of this study presented strong evidence of beneficial effect on serum albumin due to oral nutritional supplementation.

Wound complications always more frequently happen in geriatric patients and deserve more attention [31]. Our study showed that the incidence of wound complications (SSI, wound effusion) was lower in the nutritional supplementation group than in the control group. Similar results were found in previous studies [33]. A retrospective study included 10,325 joint arthroplasties, results indicated prolonged wound drainage [34]. Another study included 49,603 joint arthroplasties from a database, results demonstrated hypoalbuminemia independently predicts SSI (adjusted RR, 2.0; 95% CI, 1.5–2.8; *P* < 0.001) [35]. The decreased rate of wound complications could be related to the increased serum albumin level, which would benefit the elderly patients to maintain immunological function [13, 35].

PJI was a disaster complication for HA. Several studies investigated the association between hypoalbuminemia and PJI. A retrospective study included 30,863 joint arthroplasties (11,554 HAs included) cases and indicated that albumin had the greatest association (OR = 4.697, *P* < 0.001) with subsequent PJI [4]. Multivariable analysis showed a protective effect of nutritional supplementation in our study. The reason for the association was most likely multifactorial. Nutritional supplementation improved serum albumin level as albumin played a critical role in immune function [31]. Another reason might be that nutritional supplementation decreased the systemic inflammation following surgery by activating GLUT4 and inhibiting pro-inflammatory cell signals [36–38].

Beneficial effects are also shown on hospitalization, 30-day readmission rate, and human serum albumin infusion. These might be associated with positive effects on serum albumin level and wound complication and potentially positive on medical costs reduction.

One important issue to consider was the tolerance of oral nutritional supplementation. A few previous studies indicated an intolerance of oral nutrition such as nausea, vomiting, and diarrhea [21, 39]. We had not found any statistically significant difference of such complications between oral nutritional supplementation group and control. Unlike diarrhea, nausea and vomiting were not specific complications of intolerance, anesthetic methods, and concomitant medication (such as opioid analgesics) might also be relevant causes. The issue of oral nutritional tolerance remained to be shown.

In addition, supplementation of oral nutrition powder was potentially cost-effective. It cost about 3.4 dollars per day for oral nutrition supplementation. It was just 6% of that in serum albumin infusion, which cost 53.6 dollars per day.

The main limitation of this study was that the observation was limited to the medical records and the nutritional status was not evaluated after discharge. Even though there are many uncertain factors in the interpretation of this study, the data available seem to suggest oral supplementation of nutritional powder decreased SSI, PJI, and 30-day readmission after HA in geriatric femoral neck fracture with hypoalbuminemia. The evidence is still weak, however, and further well-designed, placebo-controlled, and double-blinded studies are needed to confirm the possible positive effects reported.

## Conclusion

HA was one of the effective surgical procedures chosen for elderly patients with femoral neck fractures. However, hypoalbuminemia was common in this population and had been identified as one of the risk factors for poor outcomes. Since hypoalbuminemia was seemed to be unmodifiable before surgery in this situation. The data in this study suggested supplementation of enteral nutritional powder after surgery as an effective protective procedure. The data indicated that postoperative nutritional supplementation could decrease surgical site infection, periprosthetic joint infection, and 30 days readmission in geriatric with hypoalbuminemia undergoing hip replacement. Therefore, postoperative nutritional supplementation for these patients should be recommended.

## Additional file

Additional file 1: **Table S1.** The formula of Enteral Nutritional Powder (TP, ENSURE®) per 100 g (DOCX 18 kb)

## Data Availability

The datasets used and/or analyzed during the current study are available from the corresponding author on reasonable request.

## Abbreviations

BMI: Body mass index
BUN: Blood urea nitrogen
CIs: Confidence intervals
COPD: Chronic obstructive pulmonary disease
DVT: Deep vein thrombosis
HA: Hip arthroplasty
MI: Myocardial infarction
Nutr. supple.: Nutritional supplementation
ORs: Odds ratios
PJI: Prosthetic joint infection
POD: Postoperative day
RCT: Randomized placebo-controlled test
SSI: Surgical site infection
TLC: Total lymphocyte count
Vit.: Vitamin
