# Supplementary material for: Supplementation of enteral nutritional powder decreases surgical site infection, prosthetic joint infection, and readmission after hip arthroplasty in geriatric femoral neck fracture with hypoalbuminemia
Source: J Orthop Surg Res. 2019 Sep 3;14:292. doi: 10.1186/s13018-019-1343-2 (PMC6724262; doi:10.1186/s13018-019-1343-2)
Supplement: Supplementary file 1 — Table S1. The formula of Enteral Nutritional Powder (TP, ENSURE®) per 100 g (DOCX 18 kb) [file 13018_2019_1343_MOESM1_ESM.docx]

**Additional file 1: Table S1. The formula of Enteral Nutritional Powder (TP, ENSURE®) per 100g**

| **Main** | | | | | | | | | | | | | | | | | | | | |
| --- | --- | --- | --- | --- | --- | --- | --- | --- | --- | --- | --- | --- | --- | --- | --- | --- | --- | --- | --- | --- |
| Energy | | | 450kcal | | | Protein | | 15.9g | | | Fat | | 15.9g | | | Linoleic acid | | | | 8.7g |
| Carbohydrate | | | 60.7g | | | Water | | 5g | | | Biotin | | 150mg | | | Choline | | | | 136mg |
| Pantothenic acid | | | | 5.0mg | | | | | | | | | | | | | | | | |
| **Vitamin** | | | | | | | | | | | | | | | | | | | | |
| Vit. A | | 1170IU | | Vit. D3 | | | 95IU | | | | | Vit. E | | 10.7IU | | | Vit. K1 | | | 18ug |
| Vit. C | | 68ug | | Folic acid | | | 200ug | | | | | Vit. B1 | | 0.72ug | | | Vit. B2 | | | 0.80ug |
| Vit. B6 | | 1.0ug | | Niacin | | | 10.0ug | | | | | Vit. B12 | | 3.1ug | | |  | | |  |
| **Minerals** | | | | | | | | | | | | | | | | | | | | |
| K | 670mg | | Na | | 360mg | | | | Cl | 610mg | | | Ca | | 230mg | | | P | 230mg | |
| Fe | 4.37mg | | Mn | | 1.2mg | | | | I | 34ug | | | Cr | | 20ug | | | Mg | 90mg | |
| Zn | 5.4mg | | Cu | | 0.52mg | | | | Se | 20ug | | | Mo | | 38ug | | |  |  | |

Vit.: vitamin.
